# Comparative Analysis of Three Trypanosomatid Catalases of Different Origin

**DOI:** 10.3390/antiox11010046

**Published:** 2021-12-26

**Authors:** Ľubomíra Chmelová, Claretta Bianchi, Amanda T. S. Albanaz, Jana Režnarová, Richard Wheeler, Alexei Yu. Kostygov, Natalya Kraeva, Vyacheslav Yurchenko

**Affiliations:** 1 Life Science Research Centre, Faculty of Science, University of Ostrava, 71000 Ostrava, Czech Republic; lubomira.chmelova@gmail.com (Ľ.C.); clarettab85@gmail.com (C.B.); amandatabitaalbanaz@gmail.com (A.T.S.A.); janna.krallova@gmail.com (J.R.); kostygov@gmail.com (A.Y.K.); luzikhina@gmail.com (N.K.); 2 Nuffield Department of Medicine, University of Oxford, Old Road Campus, Headington, Oxford OX3 7BN, UK; richard.wheeler@ndm.ox.ac.uk; 3 Zoological Institute of the Russian Academy of Sciences, 199034 St. Petersburg, Russia; 4 Martsinovsky Institute of Medical Parasitology, Tropical and Vector Borne Diseases, Sechenov University, 119435 Moscow, Russia

**Keywords:** *Vickermania ingenoplastis*, *Leptomonas pyrrhocoris*, *Blastocrithidia* sp., cyanide resistance

## Abstract

Most trypanosomatid flagellates do not have catalase. In the evolution of this group, the gene encoding catalase has been independently acquired at least three times from three different bacterial groups. Here, we demonstrate that the catalase of *Vickermania* was obtained by horizontal gene transfer from Gammaproteobacteria, extending the list of known bacterial sources of this gene. Comparative biochemical analyses revealed that the enzymes of *V. ingenoplastis*, *Leptomonas pyrrhocoris*, and *Blastocrithidia* sp., representing the three independent catalase-bearing trypanosomatid lineages, have similar properties, except for the unique cyanide resistance in the catalase of the latter species.

## 1. Introduction

Catalase (EC 1.11.1.6) is one of the most widespread enzymes in aerobic organisms [1]. Most of the typical catalases are homo-tetramers with four prosthetic heme *b* groups and, in some cases, another cofactor—NADPH [2]. This enzyme catalyzes the decomposition of hydrogen peroxide, which is produced during aerobic metabolism, into oxygen and water in a two-step process. In the first step, a ferric cation reacts with the first molecule of H_2_O_2_ producing compound I (oxidized form, oxoferryl π-cation radical) and water. In the second step, this compound reacts with the second H_2_O_2_ molecule, resulting in two single-electron reductions in the enzyme, followed by the production of oxygen and the second molecule of water [3,4]. The kinetics of this reaction depends on the distal side residues [5]. The main function of catalase is to protect cells from hydrogen peroxide, which belongs to reactive oxygen species. Although hydrogen peroxide has high activation energy and, therefore, can react only with a narrow range of biological molecules [6], in the presence of Fe^2+^ it can enter the Fenton reaction, where the final product is one of the strongest oxidants—the hydroxyl radical [7]. Catalases were studied for over 100 years [8] and experimentally determined structures of several representative enzymes (for example, those from bovine liver [9], *Penicillium vitale* [10], and *Saccharomyces cerevisiae* [11]) were published.

Phylogenetic reconstruction of the catalase evolutionary history is complicated by frequent horizontal gene transfer (HGT) events, which are especially frequent among prokaryotes [12]. The same mechanism is responsible for the presence of catalases in eukaryotes, which have acquired the corresponding genes from different sources [13,14]. The HGT is a movement of genetic material between two unrelated species in proximity, in contrast to the vertical gene transfer, where the genetic material is passed from the parent to the offspring [15,16]. The HGT drives speciation in bacteria and archaea and may easily convert a harmless species into a severe pathogen [17,18,19]. Eukaryotes most frequently have HGTs from their endosymbiotic bacteria [20,21] and viruses [22]. There are several conceptual approaches to infer HGT events in genomes [23]. One method relies on comparing genomes of closely related species or strains of the same species: the differences in gene content can indicate recent HGTs. Another approach rests on the analyses of genomic GC content and codon usage: again, the differences imply possible HGTs. Both methods are likely to underestimate the HGT events and they are not suitable to detect gene transfer between species with a similar composition of their genomes or ancient events (once integrated, the DNA progressively acquires the traits of the receiving genome). The phylogenetic approach for deducing HGTs is the most powerful of all. It is based on a comparison of the gene and organism phylogenies, and a significant discordance between them is considered conclusive evidence of HGT (reviewed in [23]).

Most eukaryotes have catalases acquired from various sources, but several groups, for instance, organisms thriving in anoxic conditions, such as *Giardia*, *Trichomonas*, *Entamoeba*, or *Cryptosporidium* spp., or those containing secondary plastids, such as euglenids or chlorarachniophytes) conspicuously lack any identifiable homolog of this gene in their genomes [14,24]. Kinetoplastid flagellates of the family Trypanosomatidae represent a case, where the distribution of catalases among lineages is mosaic [25]. The members of this family parasitize either only insects (monoxenous species) or use invertebrate vectors to shuttle between vertebrate or plant hosts (dixenous species) [26,27,28]. In this group, the catalase-encoding genes were found only in monoxenous Leishmaniinae (phylogenetic relatives of dixenous *Leishmania*—representatives of the genera *Crithidia*, *Leptomonas*, *Lotmaria* and *Novymonas*), Blastocrithidiinae (genera *Blastocrithidia* and *Obscuromonas*) and *Vickermania* spp. Importantly, catalases in these groups have been independently acquired via HGT from bacteria of different classes: Spirochaetia (Leishmaniinae) [14] and Betaproteobacteria (Blastocrithidiinae) [29], while the origin of catalase in *Vickermania* has not been investigated [30]. Interestingly, the catalase-encoding gene is absent from all the analyzed genomes of *Leishmania* spp. and their closest phylogenetic relatives—dixenous *Porcisia* and *Endotrypanum* [31], indicating that its secondary loss was apparently driven by the incompatibility of the enzyme with the dixenous life cycle of these parasites. This view was further supported by experiments with *Leishmania mexicana*, *Trypanosoma cruzi* and *T. brucei* genetically modified to express catalase [32,33,34]. In all these cases parasite development and pathogenicity were severely impaired.

In this work, we demonstrate that the catalase of *Vickermania* spp. was acquired by HGT from Gammaproteobacteria, i.e., independently from those of Leishmaniinae and Blastocrithidiinae, and provide comparative biochemical analysis of different trypanosomatid catalases of *Leptomonas pyrrhocoris*, *Blastocrithidia* sp., and *Vickermania ingenoplastis*.

## 2. Materials and Methods

### 2.1. Trypanosomatid Isolates and Cultivation

*Blastocrithidia* sp. (isolate P57), *Leptomonas pyrrhocoris* (isolate H10), and *Vickermania ingenoplastis* (isolate CP021) were cultivated as described previously [35,36,37]. Total DNA was isolated with the GeneJET Genomic DNA Purification Kit (Thermo Fisher Scientific, Waltham, USA) following the manufacturer’s protocol. The species identity was confirmed by SSU rRNA gene sequencing as in [38]. 

### 2.2. Phylogenetic Inferences

The protein sequence of *Vickermania ingenoplastis* catalase (See Data Availability section) was used as a query for a blastp search in the NCBI nr database [39]. The search resulted in over 70,000 sequences, which were then filtered as follows: *e*-value reported as 0, the hits do not contain “multispecies”, “unknown”, “partial”, “uncultured”, and “unclassified” in their identifiers, and only the first instance taken in the case of duplicates (identical sequences). The final dataset contained 11,997 sequences, including that of *V. ingenoplastis*. The sequences were aligned using MAFFT v. 7.471 [40] in automatic mode and the poorly aligned regions were removed using seqmagick v. 0.8.4 [41] with convert --squeeze-threshold of 0.5. The phylogenetic tree was built using FastTree v. 2.1.10 under LG + CAT model [42]. A subset of 20 sequences, representing the clade enclosing the sequence of *V*. *ingenoplastis* on the large tree, was selected for the phylogenetic reconstructions under the maximum likelihood criterion in IQ-Tree v. 2.1.3 [43] and by the Bayesian method in MrBayes v. 3.2.7 [44]. For the ML inference, the LG + I + G4 model was selected as the best fit by ModelFinder (implemented in IQ-TREE) based on a BIC score [45], and 1000 standard bootstrap replicates were used for the estimation of branch support. In MrBayes, we used the LG model, 400,000 generations, and all other parameters set by default. The standard deviation of split frequencies at the end of the run was below 0.01.

### 2.3. Analysis of Gene Copy Number

The catalase gene copy number was analyzed in eight trypanosomatid species with high-quality genomes available [46]: *Blastocrithidia* sp., *Crithidia bombi*, *C. expoeki*, *C. fasciculata*, *Leptomonas pyrrhocoris*, *L. seymouri*, *Novymonas esmeraldas*, and *Vickermania ingenoplastis* (Table S1). We reasoned that using lower-quality draft genomic data is not justified as it may produce artifacts, because of the substantial number of unassembled contigs. The searches were executed using tblastn and blastp with an *e*-value cut-off of 10^−10^, using the catalase of *L. pyrrhocoris* as a query. The catalase clade assignment was done using the NCBI-CDD search tool [47,48]. The protein sequences were aligned using MAFFT v. 7.471 with the L-INS-i iterative refinement method and the average protein identity within the alignment was assessed using the esl-alistat script v. 0.46 from the HMMER package [49]. A pairwise identity matrix was calculated using Clustal Omega v. 2.1 [50] and visualized using DisplayR v. 1.0.1 [51]. 

### 2.4. Sequence Analysis of Catalase

The protein sequences of *Homo sapiens* (NP_001743), *L. pyrrhocoris* (XP_015656183), and *Blastocrithidia* sp. (QDL90315) catalases were downloaded from NCBI and aligned with that of *V. ingenoplastis* by MAFFT v. 7.471 using the G-INS-i iterative refinement method. The presence of conserved domains was analyzed with the NCBI-CDD tool. 

### 2.5. Expression and Purification of Recombinant Catalases

The *L. pyrrhocoris* and *V. ingenoplastis* catalase ORFs were amplified from genomic DNA using specific primers containing *Nde*I and *Not*I restriction sites (Table S2). Because *Blastocrithidia* sp. genomic sequence contains in-frame stop codons [52], it was modified to preserve the translated amino acid identity (Figure S1) and synthesized by Eurofins Genomics (Luxembourg). DNA fragments were cloned into the pET42b+ expression vector (MilliporeSigma, Burlington, NJ, USA) following *Nde*I and *Not*I digestion. The resultant plasmids were transformed into the ArcticExpress (DE3) RIL *Escherichia coli* (Agilent Technologies, Santa Clara, CA, USA).

Proteins were expressed and purified on Ni Sepharose 6 Fast Flow resin (GE Healthcare, Chicago, IL, USA) per the manufacturer’ instructions. After elution with 150–300 mM imidazole, the samples were dialyzed overnight at 4 °C against 50 mM potassium phosphate buffer pH 7.0. The purified protein was either used directly for enzymatic assays or preserved in 20% (*v*/*v*) glycerol at −80 °C. 

### 2.6. Catalase In-gel Activity Staining

Purified proteins (10 μg) and bovine liver catalase (5 μg, MilliporeSigma), used as a standard, were separated in 6% polyacrylamide gel, pH 8.8 under non-denaturing conditions at 4 °C. After separation, the gel was washed thrice with ultrapure water and incubated with 0.3% hydrogen peroxide for 10 min at room temperature. The staining components (2% FeCl_3_ and 2% K_3_[Fe(CN)_6_], both *w*/*v*) were prepared freshly and filtered through a 0.22 μm filter before use. They were added to the gel and the presence of an achromatic band indicated the catalase activity [53].

### 2.7. Catalase Activity Assays

The activity of catalase was measured spectrophotometrically using DU-730 UV–Vis spectrophotometer (Beckman Coulter, Brea, CA, USA) at 240 nm. The reactions (1 mL) comprised 50 mM potassium phosphate buffer pH 7.0, ~300 ng (1 nM) enzyme and various concentrations of a substrate (from 0.5 to 75 mM). The concentration of decomposed hydrogen peroxide was determined using ε_240_ of 43.6 M^−1^cm^−1^ [54]. One unit (U) of activity was defined as the amount of the enzyme that reduces 1 μmol of hydrogen peroxide per 1 min. All measurements were performed in triplicates. The enzymatic properties were calculated using non-linear regression analysis [55] in Prism v9.2.0 (GraphPad Software, San Diego, CA, USA). In short, the enzyme velocity (µmoles/min) at a given concentration was calculated from 15 measurements and used to deduce K_M_ and V_MAX_ values and standard deviation. The observed values were calculated from the Michaelis–Menten plots of velocity over concentration: three independent biological replicates were averaged and used for calculating the observed K_M_ and V_MAX_ values [56]. 

The pH optimum of catalases was measured with 15 mM H_2_O_2_ and buffers with different pH—sodium citrate (pH 5), potassium phosphate (pH 6–8), Tris-HCl (pH 9), and Glycine-NaOH (pH 10–11). The ionic strength of all buffers was kept at 50 mM. All measurements were performed in triplicate.

The enzymatic inhibition was measured with 15 mM H_2_O_2_ and 50 mM potassium phosphate, pH 7. The enzyme was incubated with different concentrations of inhibitors (KCN or 3-amino-1,2,4-triazole, hereafter denoted as 3-AT, both from MilliporeSigma) for one minute, then the substrate was added and the residual activity was measured at 240 nm for 2 min [56]. All measurements were performed in triplicate.

### 2.8. Protein Structure Prediction

The structures of the three catalases under study were predicted by the ColabFold implementation of AlphaFold 2 [57,58] with AlphaFold parameters from 2021-07-14, not using Amber relaxation or PDB templates. The source sequences databases used were UniREF, BDF, Uniclust, MGNify, supplemented with an additional database specifically gathered for Kinetoplastid species, as previously described [59]. The structures were visualized in PyMOL v. 2.3.0 [60]. The heme group was added to the catalase structures of *V. ingenoplastis*, *L. pyrrhocoris*, and *Blastocrithidia* sp. by superimposition on those of their closest relatives identified by BLAST (*Acinetobacter* sp. (PDB: 6PT7), *Vibrio salmonicida* (PDB: 2ISA), and *Pseudomonas aeruginosa* (PDB: 4E37), respectively) using the ‘cealign’ function in PyMOL. The RMSD values were calculated in PyMOL.

## 3. Results

### 3.1. Phylogenetic Inferences, Analyses of Sequences and Gene Copy Number

**Figure 1:**
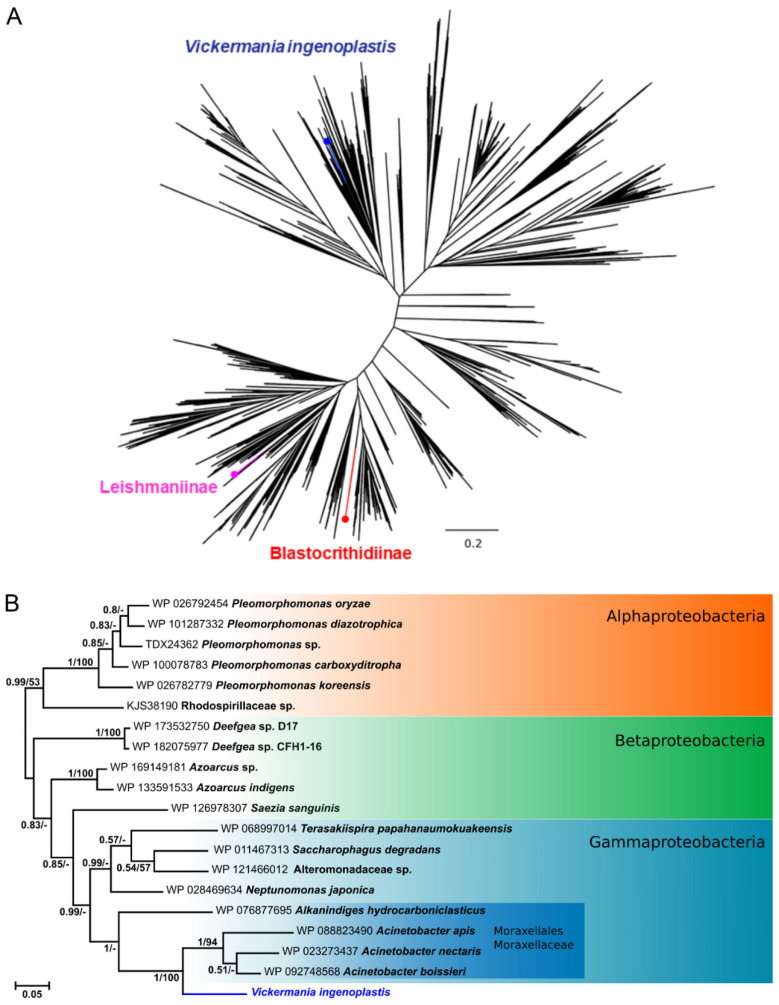
Origin of catalases in trypanosomatids. (**A**) Phylogenetic tree showing the position of trypanosomatid enzymes. (**B**) Phylogenetic tree of the closest relatives of the catalase in *V. ingenoplastis*. Numbers indicate Bayesian posterior probability and bootstrap supports, respectively. Bacterial taxonomy (classes for all, order, and family for the closest relatives of *V. ingenoplastis*) is on the right. Scale bars in (**A**,**B**) indicate the number of substitutions per site.

The phylogenetic analysis of the catalase-encoding gene showed a remarkable divergence of this enzyme within the family Trypanosomatidae (Figure 1A). In agreement with previous studies, the catalases of Leishmaniinae and Blastocrithidiinae clustered with those of spirochetes and betaproteobacteria, respectively [29]. The catalase of *Vickermania ingenoplastis* was revealed to be most closely related to that of *Acinetobacter* spp. of the family Moraxellaceae belonging to the order Moraxellales of the class Gammaproteobacteria (Figure 1B). The statistical support of this relationship is absolute by both methods used, even though, in general, the resolution of the tree is rather low. Another representative of the family Moraxellaceae (*Alkanindiges*) is the next closest relative. This places Gammaproteobacteria as a new source of this enzyme for trypanosomatids. 

Of note, among bacteria, catalases are often acquired by HGT and one species of bacteria may even contain enzymes of different origin [61]. The tree in Figure 1B contains 19 closest relatives of *V. ingenoplastis* catalase, of which only eight belong to Gammaproteobacteria, despite the fact that the dataset used here contained 4419 catalase sequences from this bacterial class. 

Using the NCBI-CDD search tool we predicted conservative domains of the three trypanosomatid catalases (*Blastocrithidia* sp. P57, *Leptomonas pyrrhocoris*, and *Vickermania ingenoplastis*). The newest “kid on the block”, a catalase of *V. ingenoplastis*, like the other trypanosomatid catalases, belongs to the clade 3 (IPR040333), the most abundant subfamily found in all the kingdoms [1]. These enzymes are relatively small varying in size between 43 to 75 kDa, bind the protoheme IX (heme b), require NADPH as a second redox-active cofactor, and form tetramers.

**Figure 2:**
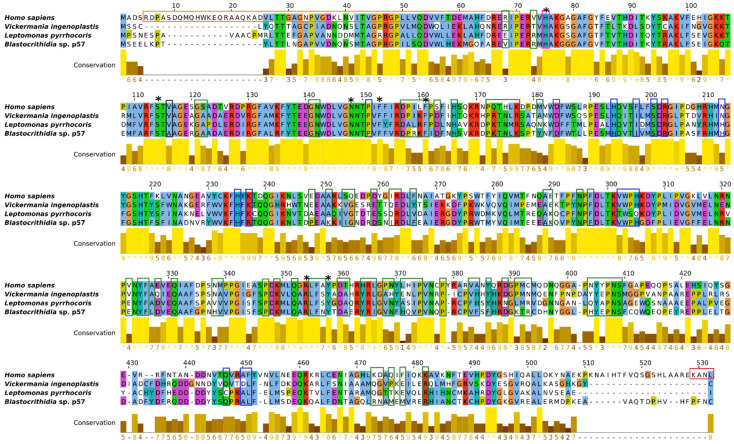
Multiple alignment of catalase amino acid sequences for three trypanosomatids and well-studied *Homo sapiens* (selected as a reference). Predicted domains and sites: black asterisk, heme binding pocket; blue box, NADPH binding site; green box, tetramer interface; orange box, N-terminal threading arm; pink box, distal histidine binding site for 3-AT inhibitor; red box, peroxisomal targeting signal. The Val116 is boxed in black. Conservation color below alignment follows the ClustalX scheme. The numerical index indicates the level of conservation for each column of the alignment. The score is shown below the histogram, with higher score denoting higher level of conservation. The conserved positions with a score of 11 are indicated by yellow asterisks. Positions with a score of 10 (possessing mutations but retaining all physicochemical properties) are marked by ‘+’.

The catalase sequences in general and those of trypanosomatids are fairly well conserved (Figure 2). Amino acid residues forming the heme binding pocket are invariant (marked by black asterisks). The most variable is the sites responsible for oligomer formation (boxed in green). The His75 (hereafter, the amino acid numbering refers to the human catalase, shown on top in Figure 2, unless specified otherwise), which has been shown to covalently bind 3-AT [62], is conserved in trypanosomatid enzymes (boxed in pink). Strikingly, the Val116, which is invariably conserved in all previously investigated catalases and appearing to be crucial for the enzyme [2], in *Blastocrithidia* sp. p57 has been substituted with Ala (Val99Ala).

**Figure 3:**
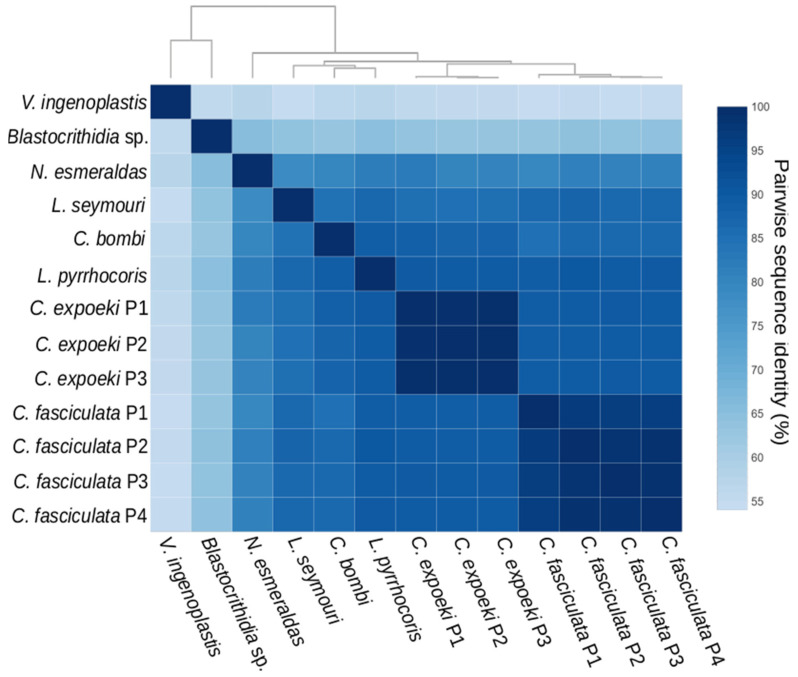
Heatmap for the pairwise sequence identity percentage at the amino acid level between 13 catalase sequences. The color brightness reflects the identity percentage. A dendrogram on top visualizes the result of hierarchical clustering calculations. Full species names and sequence IDs are listed in Table S1. The source data for this diagram are in Table S3.

We noticed that, while being present as a single copy in genomes of *Blastocrithidia* sp., *C. bombi*, *L. pyrrhocoris*, *L. seymouri*, and *V. ingenoplastis*, the catalase-encoding gene has been multiplicated in the genomes of at least two *Crithidia* spp.–*C. expoeki* and *C. fasciculata* containing 3 and 4 nearly-identical paralogs, respectively (gene IDs C_expoeki_000029100, C_expoeki_000005110, C_expoeki_160005000 and CFAC1_250006200, CFAC1_160031400, CFAC1_280006600, CFAC1_290005500 respectively) (Figure 3, Table S3). In trypanosomatids, the amplification of a gene is a known mechanism of increasing its expression [63,64]. The reason why these particular species may require higher catalase expression levels remains to be investigated further.

### 3.2. Biochemical Characterization of Trypanosomatid Catalases

**Figure 4:**
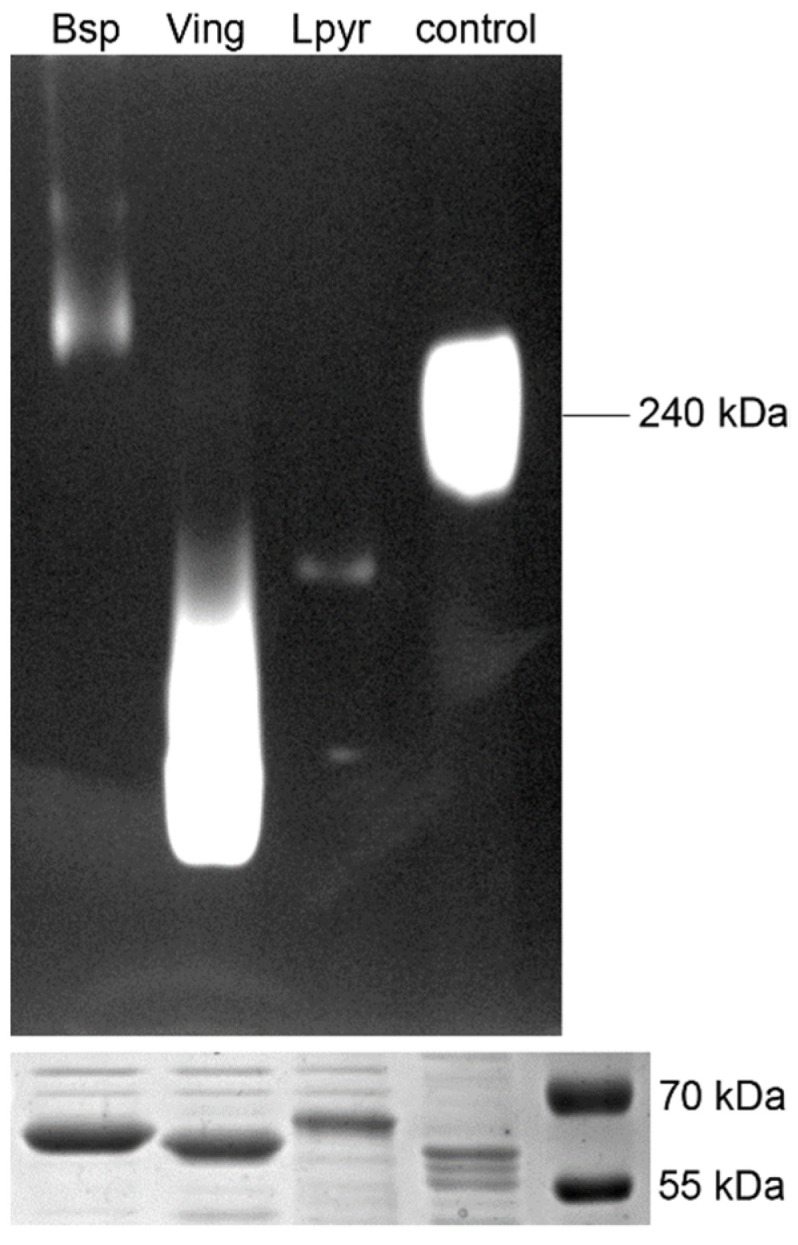
In-gel activity of the purified catalases of *Blastocrithidia* sp. P57 (Bsp), *Vickermania ingenoplastis* CP21 (Ving), and *Leptomonas pyrrhocoris* H10 (Lpyr). The bovine liver catalase (homo-tetramer of approximately 240 kDa, lane 4) served as a control. Ten and five micrograms were assayed for trypanosomatids’ and bovine enzymes, respectively. The loading control (SDS-PAGE) is shown below.

The C-terminally His-tagged catalases of *Blastocrithidia* sp. P57, *Leptomonas pyrrhocoris* H10, and *Vickermania ingenoplastis* CP21 were expressed in *E. coli* BL21(DE3)pLysS at different temperatures in the range between 16 and 37 °C. In all these cases, the majority of the recombinant protein was found in the inclusion bodies, implying a mismatch between the rate of protein synthesis and the capacity of cells to fold them into their native state [65]. Therefore, we switched to the ArcticExpress (DE3) RIL *E. coli* and expression at 10 °C. In these conditions, the amount of soluble protein was substantially higher than in other *E. coli* strains tested previously. The calculated size of the His-tagged catalase monomer is 55.4 kDa, 54.9 kDa, and 56.4 kDa for *L. pyrrhocoris*, *V. ingenoplastis*, and *Blastocrithidia* sp., respectively. These numbers correlated well with the protein sizes observed on the SDS-PAGE (Figure 4, bottom panel). The enzymatic activity of purified catalases was confirmed by in-gel activity staining using bovine liver catalase as a control (Figure 4, upper panel). We noticed that catalases of the analyzed trypanosomatid species run differently on the native gel, implying differences in the composition or stability of their multimeric complexes. 

Next, we investigated the kinetic properties of the three trypanosomatid enzymes. Generally speaking, catalases do not follow the classical Michaelis–Menten kinetics over the whole range of substrate concentration: (i) substrate inhibition is observed at high concentrations of H_2_O_2_; (ii) the reaction has two enzymatic steps [3]. However, at low hydrogen peroxide concentrations, the Michaelis–Menten kinetics can be approximated and K_M_ and V_MAX_ values calculated. For measuring the kinetic parameters of purified catalases, we employed a continuous assay that directly monitors the decrease of hydrogen peroxide concentration over time at 240 nm. Due to the formation of bubbles that could interfere with spectrophotometric measurements, we kept the total amount of catalase in the reaction at approximately 300 ng (~1.33 nM). The kinetics data (observed and calculated by non-linear regression analysis K_M_ and V_MAX_) are presented in Table 1. The K_M_ for *L. pyrrhocoris* and *V. ingenoplastis* catalase are similar (9.40 ± 1.39 mM and 8.14 ± 0.90 mM), while this value for the *Blastocrithidia* sp. catalase is higher (22.8 ± 2.5 mM). The observed trend for V_MAX_ is the same. As was described before, the catalases exhibit substrate inactivation [56]. The catalase of *L. pyrrhocoris* H10 started to be inactivated when the concentration of substrate reached 35 mM. For other catalases, the concentration of their substrate needed for inactivation was 45 mM and 50 mM for *V. ingenoplastis* and *Blastocrithidia* sp., respectively (Figure S2).

We also tested two compounds that are known to inhibit the catalase activity: (1) a reversible competitive inhibitor KCN acting as a sixth ligand of the iron in the heme prosthetic group and featuring linear binding [66], and irreversible 3-AT forming covalent non-coplanar adduct by reacting with a distal histidine of catalase [62]. For all the investigated enzymes we measured the inhibitor concentration necessary to half the specific activity, IC_50_ (Table 2). 

All catalases reacted similarly to the presence of 3-AT with IC_50_ ranging between 10 and 16 mM. However, the KCN sensitivity varied greatly from 4.34 μM in the case of the most sensitive enzyme from *V. ingenoplastis* to over 1200 μM for the catalase of *Blastocrithidia* sp. (Figure S3).

To further characterize the purified enzymes, we tested their pH optima. The activity of catalases was assayed at a pH ranging from 5 to 11. The enzymes were not active at low pH. The pH optimum for *L. pyrrhocoris* catalase was around 7, while at higher pH its specific activity decreased. The graphs for *V. ingenoplastis* and *Blastocrithidia* sp. catalases had two peaks at ~pH 7 and 9, and pH 6 and 11, respectively (Figure S4). 

### 3.3. Structural Insight into Trypanosomatid Catalases

**Figure 5:**
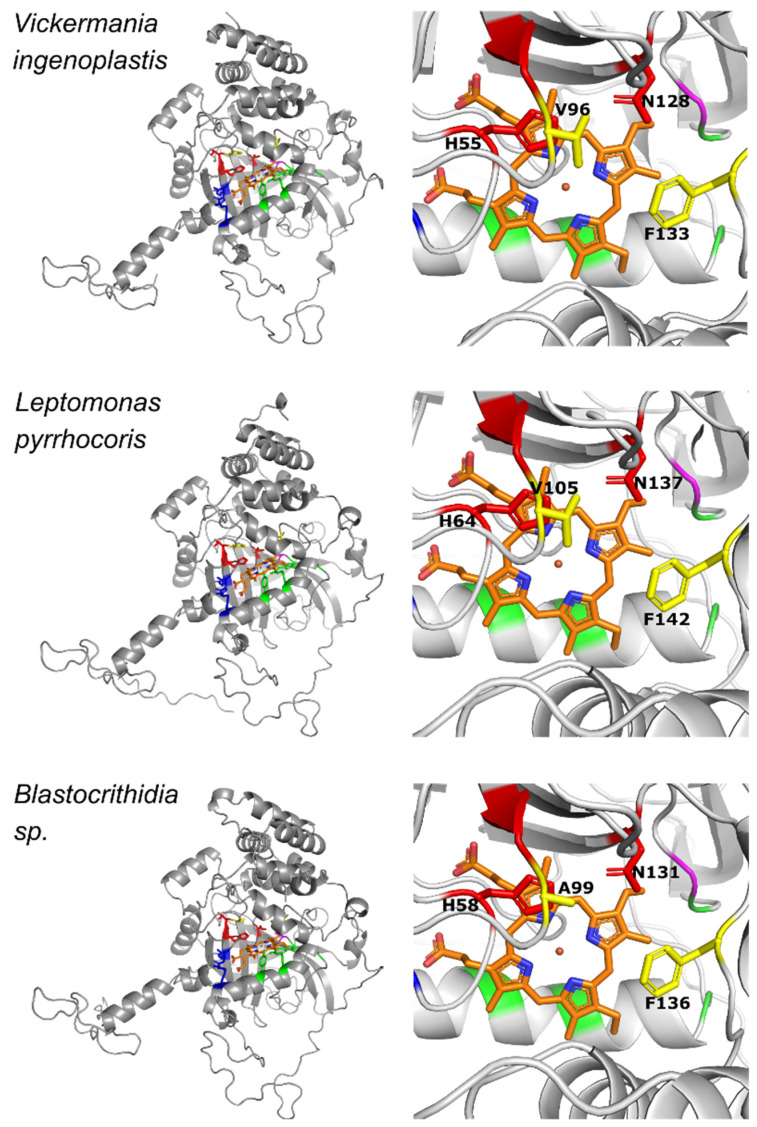
Predicted three-dimensional structures of the monomers of trypanosomatid catalases. Left column: overall structure; right column: heme pocket and the innermost part of the channel. Superimposed heme group is in orange with N and O atoms colored in blue and pink, respectively. Green and red indicate proximal and distal heme sides, respectively. Blue, heme propionate side chains; magenta, heme vinyl side chains; residues shown in red and yellow are His and Asn of the distal side and Ala/Val and Phe of the main channel, respectively. The PAE (predicted average error) plots for the modeled structures are presented in Supplementary Figure S5.

Prompted by the observation that the catalase of *Blastocrithidia* sp. is resistant to cyanide, we investigated this further by comparing predicted structures of the three trypanosomatid catalases (Figure 5). Predictions were made using AlphaFold 2 with high confidence (pLDDT and pAE almost entirely over 90 and below 5 Å, respectively, Figure S5). The inferred structures showed high overall similarity (Figure 5, left panel) and even higher similarity for the catalytic center (Figure 5, right panel). Furthermore, we calculated root-mean-square deviation of atomic positions (RMSD) values and compared them between the three trypanosomatid catalases and their closest PDB relatives (6PT7, 2ISA, 4E37) (Table S4). All the estimated values were in the same range, further supporting a note of the high similarity between these enzymes. 

To the best of our knowledge, all previously studied monofunctional catalases are cyanide-sensitive [56], making the enzyme of *Blastocrithidia* sp. truly unique. As mentioned above, the cyanide acts as a competitive inhibitor in binding as a sixth ligand of the iron in the heme prosthetic group. This process takes place in the heme binding pocket. Both the substrate and the inhibitor must gain access to the deeply buried active site of the catalase. The main channel approaches the heme pocket in perpendicular orientation to the plane of heme [67]. The first part of the channel in small-subunit catalases is funnel-shaped, making it accessible to bigger molecules. The second part of the channel contains well-conserved amino acid residues that restrict the passage of bigger molecules with van der Waals diameter over 3.5 Å [56,68]. Previous studies revealed that the main channel is also important for the proper orientation of the substrate [69,70]. Direct comparison of the predicted structures for *Leptomonas pyrrhocoris*, *Vickermania ingenoplastis*, and *Blastocrithidia* sp. catalases revealed overall structure conservation (Figure 5, left column), confirming previous sequence analysis (Figure 2). Nevertheless, we detected changes in the amino acids forming the main channel (Figure 5). The most striking one was the mutation of the highly conserved Val99 into Ala in the enzyme of *Blastocrithidia* sp. Notably, this mutation is preserved in another previously analyzed species of *Blastocrithidia*, *B. triatomae* [29]. In *S. cerevisiae*, the Val116Ala substitution caused the increase in peroxidatic activity of the catalase-A [11]. A similar change in the hydroperoxidase II of *E. coli* caused a decrease in its enzymatic activity [71]. It has been proposed that dimensions of the channel might present important determinants of the rate for H_2_O_2_ or inhibitors’ movement into the active site [72]. To investigate this, we measured the constriction of the main channel (narrowest poInt. between Phe136 and Ala99, Phe142 and Val105, Phe133 and Val96 for *Blastocrithidia* sp., *L. pyrrhocoris*, and *V. ingenoplastis*, respectively). This value was the highest for *Blastocrithidia* sp. (Figure 5, right column), indicating that Val to Ala substitution results in the main channel enlargement, potentially making it more accessible to water. 

## 4. Discussion

In this work, we focused on catalases of trypanosomatids and demonstrated that in all cases known thus far, these enzymes have been independently acquired by Leishmaniinae, Blastocrithidiinae, and *Vickermania* via HGT from bacteria of the classes Spirochaetia, Betaproteobacteria, and Gammaproteobacteria (Figure 1). The putative HGT scenarios for Leishmaniinae and Blastocrithidiinae have been discussed in earlier works [14,29] and are linked to the midgut dwelling, which is typical for many members of the former and all of the latter subfamily [73]. The same logic applies to the Gammaproteobacteria of the family Moraxellaceae acquired by *Vickermania*. The putative HGT must have occurred in the fly midgut that can be coinhabited by both organisms [36,74]. The more intriguing question is why the catalase was acquired only by some trypanosomatids, while the vast majority “live happily” without it? Insects use H_2_O_2_ to control their gut microflora and bacteria survival depends on the presence of catalase [75,76]. However, most trypanosomatids rely on glutathione peroxidases and peroxiredoxins, and even the flagellates that acquired catalase still preserve these enzymes [77], making the whole story especially mysterious. 

Usually, catalases are tetrameric [1], but other modes of complex organization, for example, enzymatically active oligomers, have been also documented [78,79]. Our data suggest that the recombinant catalases of *Leptomonas pyrrhocoris* and *Vickermania ingenoplastis* are not tetrameric. However, it cannot be excluded that tetramerization in these two species requires posttranslational modifications, which could not be achieved when expressing enzymes in *E. coli*. 

Two out of three investigated trypanosomatid catalases showed cyanide sensitivity (IC_50_ of 4.3 and 51.5 µM for *V. ingenoplastis* and *L. pyrrhocoris*, respectively) comparable to that of other previously tested enzymes (9–300 µM) [56]. However, the catalase of *Blastocrithidia* sp. was not sensitive to any concentration applied, making this enzyme truly unique. To date, this property has been associated only with some manganese-dependent enzymes, known as pseudocatalases [80]. The lack of sensitivity to cyanide inhibition in the case of *Blastocrithidia* enzyme may be a result of steric effects, the ability of distal channel residues to stabilize the formation of hydrogen bonds, electrostatic or van der Waals interactions. Additional structural analyses by X-ray crystallography, NMR, Raman spectroscopy, or cryoelectron microscopy could shed light on the differences between catalases in Trypanosomatidae.

The analyzed catalases showed a broad pH optimum from pH 7 to 9. This range is similar to that of other monofunctional catalases [81,82,83]. The activity significantly decreased at low pH, which is explained by the heme dissociation [84].

The final poInt. we would like to comment on concerns K_M_ and V_MAX_ calculations. The often-adopted approach relies on the use of Lineweaver–Burk double reciprocal plot to estimate kinetic parameters of the enzymes [85]. The obtained results may substantially differ from the experimental ones; for example, the calculated and observed K_M_ values for the *Listeria seeligeri* catalase were 111 and 49 mM, respectively [56]. Similarly, when K_M_ for *L. pyrrhocoris* catalase was calculated using this method, the obtained value of 105 mM did not correspond to the observed one of 14mM. Therefore, in this work, we employed another approach (based on the non-linear regression analysis), which resulted in comparable calculated and observed values (Table 1).

## 5. Conclusions

In conclusion, here we demonstrated that catalases of Trypanosomatidae have different origins (from spirochetes, beta- and gammaproteobacteria) and exhibit different biochemical properties, despite the high similarity of their structures. The most striking finding is the cyanide resistance of the *Blastocrithidia* sp. enzyme, which is unprecedented for classical heme-dependent catalases. 

## Figures and Tables

**Table 1:** Observed and calculated by non-linear regression analysis V_MAX_ and K_M_ values of trypanosomatid catalases expressed (in μM/min and mM, respectively).

Catalase	V_MAX_ Calculated	V_MAX_ Observed	K_M_ Calculated	K_M_ Observed
*L. pyrrhocoris*	3077 ± 97	3448	9.40 ± 1.39	13.95
*V. ingenoplastis*	3890 ± 316	3922	8.14 ± 0.9	9.96
*Blastocrithidia* sp.	4543 ± 158	4036	22.80 ± 2.5	24.63

**Table 2:** KCN and 3-AT IC_50_ values for trypanosomatid catalases (in µM and mM, respectively).

Catalase	KCN	3-AT
*L. pyrrhocoris*	51.5	10.40
*V. ingenoplastis*	4.34	11.43
*Blastocrithidia* sp.	>1200	15.59

## Data Availability

The sequences of *Vickermania ingenoplastis* and *Blastocrithidia* sp. catalases are available from the NCBI GenBank (https://www.ncbi.nlm.nih.gov/genbank/, accessed on 1 November 2021) under accession numbers BK059484 and MK934828, respectively. Catalase sequences of *Leptomonas pyrrhocoris* is available from the TriTrypDB (https://tritrypdb.org/tritrypdb/, accessed on 1 November 2021) under accession number LpyrH10_15_0020.

## Supplementary Materials

The following are available online at https://www.mdpi.com/article/10.3390/antiox11010046/s1, Figure S1: DNA and conceptually translated sequence of *Blastocrithidia* sp. catalase. The in-frame stop codons were converted into sense codons (highlighted in yellow) preserving the amino acid sequence (on top); Figure S2: Michaelis–Menten plots of catalase activity with increasing substrate concentration. Data for *L. pyrrhocoris*, *V. ingenoplastis*, and *Blastocrithidia* sp. are shown in panels (a), (b), and (c), respectively. The red dot indicates a measurement out of range; Figure S3: Kinetic analysis of catalase activity in the presence of inhibitors; Figure S4: Kinetic analysis of catalase activity at different pH; Figure S5: PAE plots for structures of catalases predicted in this study; Table S1: Source of genomic data used for catalase copy number analysis; Table S2: Sequences of primers used in this work; Table S3: Pairwise sequence identity values for 13 trypanosomatid catalases; Table S4: RMSD values for the three trypanosomatid catalases and their closest PDB relatives. Data are presented in angstroms.
