# Supplementary material for: Comparative Analysis of Three Trypanosomatid Catalases of Different Origin
Source: Antioxidants (Basel). 2021 Dec 26;11(1):46. doi: 10.3390/antiox11010046 (PMC8773446; doi:10.3390/antiox11010046)
Supplement: Supplementary file 1 [file antioxidants-11-00046-s001.zip › Figure S1.pdf]

Figure S1: DNA and conceptually translated sequence of *Blastocrithidia* sp. catalase. The in-frame stop codons were converted into sense codons (highlighted in yellow) preserving the conserved amino acid sequence (on top)

```
1     M  S  E  E  L  K  P  T  Y  L  T  T  L  N  G  A  P  V  V  D
1     ATGTCAGAGGAATTGAAGCCTACCTATCTCACCACCCTCAATGGTGCACCTGTGGTGGAT
21    N  Q  N  S  M  T  A  G  P  R  G  P  I  L  S  Q  D  V  W  L
61    AATCAAAACAGCATGACAGCAGGACCACGTGGTCCGATCTTGTCGCAGGATGTGTGGTTA
41    H  E  K  M  G  Q  F  A  R  E  V  I  P  E  R  R  M  H  A  K
121   CATGAGAAGATGGGACAGTTTGCACGTGAGGTGATCCCAGAGCGTCGAATGCATGCTAAG
61    G  G  G  A  F  G  V  F  T  V  T  H  D  I  T  K  Y  T  R  A
181   GGTGGTGGTGCGTTTGGTGTGTTTACCGTCACTCATGACATCACTAAGTACACTCGGGCA
81    K  L  F  S  E  I  G  K  Q  T  E  M  F  A  R  F  S  T  A  A
241   AAATTGTTTAGTGAAATTGGTAAACAAACCGAAATGTTTGCCCGCTTTTCCACAGCAGCC
101   G  E  R  G  A  A  D  A  E  R  D  I  R  G  F  A  L  R  F  Y
301   GGAGAGCGCGGTGCTGCGGATGCGGAGCGTGACATTCGTGGTTTTGCCCTTCGCTTCTAC
121   T  E  E  G  N  W  D  L  V  G  N  N  T  P  V  F  F  V  R  D
361   ACCGAAGAAGGGAATTGGGATTTGGTGGGCAACAACACACCAGTTTTCTTTGTCCGTGAC
141   P  R  K  F  I  D  F  N  H  S  V  K  R  D  P  K  T  N  L  K
421   CCGCGCAAGTTTATTGACTTCAATCATTCTGTCAAGCGTGATCCTAAAACAAATTTGAAA
161   S  P  T  Y  N  F  D  F  W  T  L  L  P  E  S  M  H  Q  V  T
481   AGTCCAACATACAACTTTGACTTTTGGACCTTGTTGCCGGAATCTATGCACCAAGTAACT
181   I  V  M  S  D  R  G  I  P  A  S  F  R  H  M  H  G  F  G  S
541   ATTGTAATGTCTGATCGAGGCATTCCAGCATCATTTCGTCACATGCACGGCTTCGGAAGT
201   H  T  F  S  F  I  N  A  D  N  V  R  Y  W  V  K  F  H  F  R
601   CACACATTTAGCTTTATCAATGCAGATAATGTGAGATACTGGGTAAAGTTTCATTTTCGC
221   T  Q  Q  G  I  K  N  L  T  D  P  E  A  K  K  I  I  G  N  D
661   ACTCAACAAGGTATAAAAAACTTGACAGACCCTGAGGCTAAAAAAATAATTGGTAATGAC
241   R  D  S  N  I  R  D  L  F  E  A  I  E  R  G  D  Y  P  R  W
721   CGTGACAGCAACATACGTGACTTGTTTGAGGCAATTGAGCGAGGTGATTACCCTCGTTGG
261   T  M  Y  V  Q  I  M  T  E  E  E  A  K  Q  V  P  Y  N  P  F
781   ACGATGTATGTGCAAATTATGACCGAGGAGGAGGCAAAACAGGTGCCTTACAATCCATTT
281   D  L  T  K  V  W  P  H  G  D  F  P  L  I  E  V  G  F  F  E
841   GACTTGACCAAAGTGTGGCCTCATGGCGATTTTCCGTTAATCGAGGTCGGCTTTTTCGAA
301   L  N  R  N  P  E  N  Y  F  L  D  V  E  Q  A  A  F  G  P  N
901   CTCAATCGCAATCCTGAAAATTACTTCCTTGATGTTGAACAAGCTGCCTTTGGGCCTAAC
321   H  V  V  P  G  I  S  F  S  P  D  K  M  L  Q  A  R  L  F  N
961   CACGTCGTGCCAGGCATCAGCTTTTCACCTGATAAAATGTTACAGGCACGTTTGTTTAAC
341   Y  T  D  A  E  R  Y  R  I  G  V  N  F  H  Q  V  P  V  N  Q
1021  TATACAGACGCAGAACGTTATCGTATTGGTGTAAATTTTCATCAAGTACCAGTAAATCAG
361   P  R  C  P  V  F  S  F  H  R  D  G  K  T  R  C  D  H  N  Y
1081  CCACGGTGTCCTGTATTCTCGTTTCATCGTGATGGTAAGACGCGATGTGATCACAACTAT
381   G  G  L  P  H  Y  E  P  N  S  F  C  Q  W  Q  E  Q  P  E  Y
1141  GGTGGATTACCACATTACGAGCCTAATAGCTTTTGTCAATGGCAGGAACAGCCAGAGTAT
401   R  E  P  P  L  E  L  T  G  D  A  D  F  Y  D  F  R  Q  D  D
1201  CGTGAGCCACCACTGGAGTTGACTGGTGATGCAGATTTTTACGATTTCAGACAAGACGAC
421   D  D  Y  Y  S  Q  P  R  A  L  F  L  L  M  S  D  E  Q  K  Q
1261  GACGATTACTACTCTCAACCAAGAGCATTGTTTTTGTTAATGAGCGATGAACAGAAACAA
441   A  L  F  D  N  T  A  G  Q  L  R  N  A  M  E  M  V  R  E  R
1321  GCGTTGTTCGACAACACAGCAGGTCAATTGCGTAATGCTATGGAAATGGTCCGTGAGCGT
461   H  I  A  N  C  T  K  C  H  P  D  Y  G  K  G  V  R  E  A  L
1381  CACATTGCCAACTGCACTAAATGTCATCCTGATTATGGCAAAGGTGTGCGTGAGGCACTT
481   E  R  M  D  P  K  E  A  V  A  Q  T  D  P  H  V  H  F  P  F
1441  GAACGAATGGATCCTAAAGAGGCCGTCGCCCAAACAGACCCGCATGTTCACTTCCCTTTT
501   N  C  *
1501  AATTGTTAA
```
