# Supplementary material for: Comparative Analysis of Three Trypanosomatid Catalases of Different Origin
Source: Antioxidants (Basel). 2021 Dec 26;11(1):46. doi: 10.3390/antiox11010046 (PMC8773446; doi:10.3390/antiox11010046)

Figure S2. Michaelis-Menten plots of catalase activity with increasing substrate concentration.
The red dot indicates a measurement out of range.

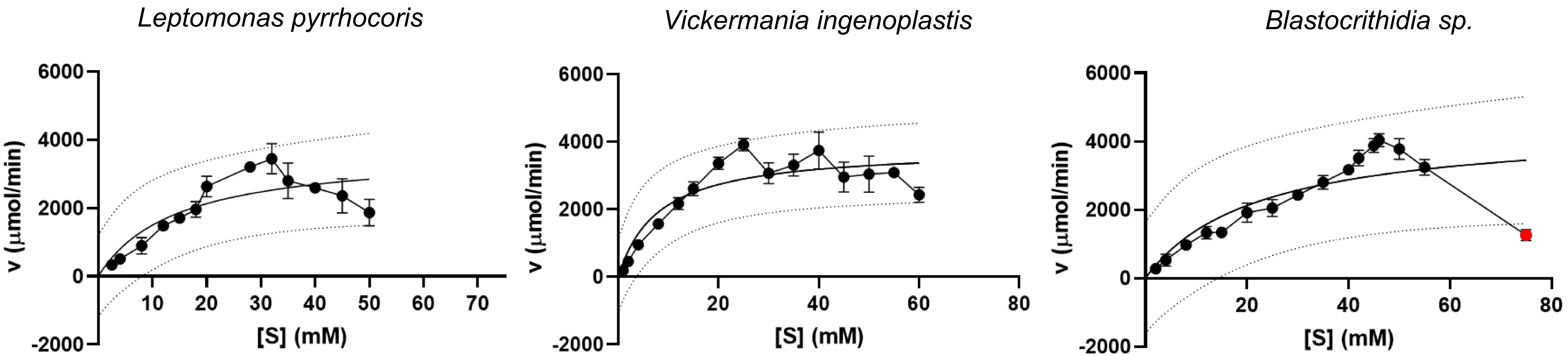

Supplement: Supplementary file 1 [file antioxidants-11-00046-s001.zip › Figure S2.pdf]
