# Supplementary figures and images for: Comparative Analysis of Three Trypanosomatid Catalases of Different Origin
Source: Antioxidants (Basel). 2021 Dec 26;11(1):46. doi: 10.3390/antiox11010046 (PMC8773446; doi:10.3390/antiox11010046)

Figure S3: Kinetic analysis of catalase activity in the presence of inhibitors

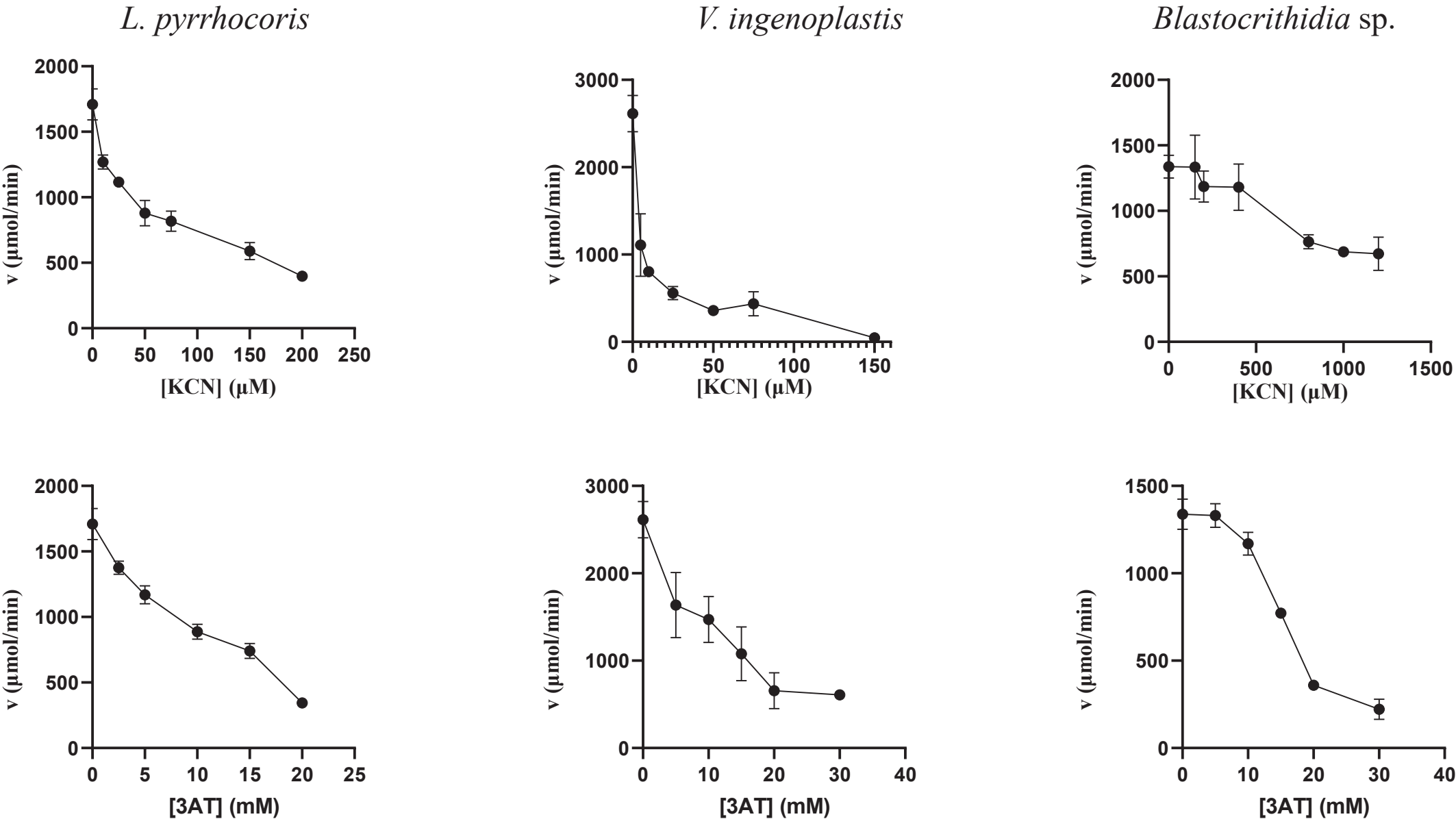

Supplement: Supplementary file 1 [file antioxidants-11-00046-s001.zip › Figure S3.pdf]

Figure S5: PAE plots for structures of catalases predicted in this study

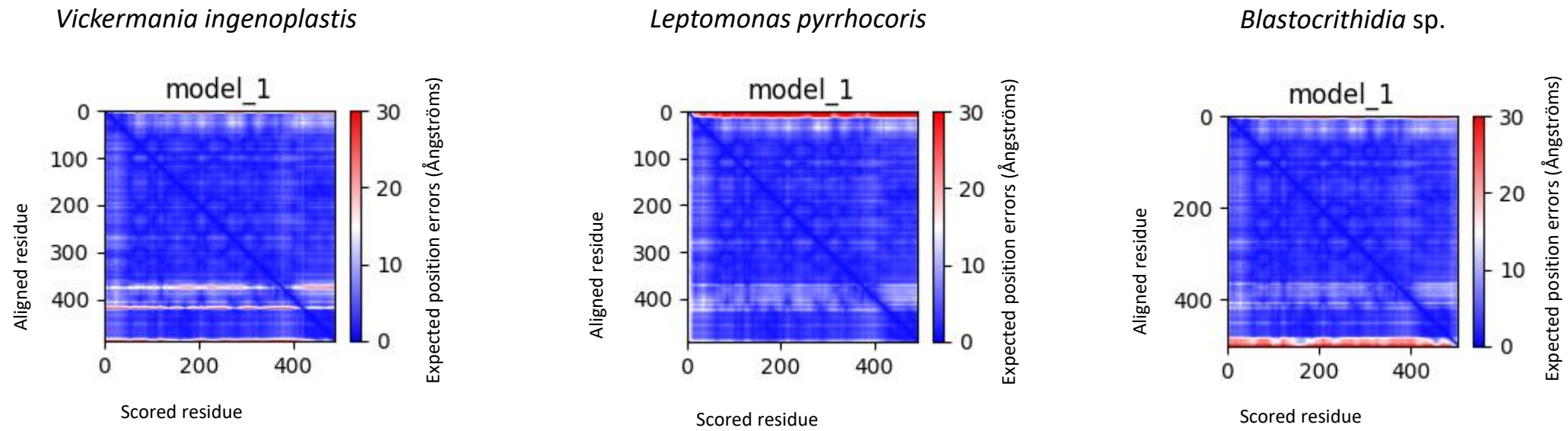

Supplement: Supplementary file 1 [file antioxidants-11-00046-s001.zip › Figure S5.pdf]
